# Prevalence of Mindfulness Practices in the US Workforce: National Health Interview Survey

**DOI:** 10.5888/pcd14.160034

**Published:** 2017-01-05

**Authors:** Diana Kachan, Henry Olano, Stacey L. Tannenbaum, Debra W. Annane, Ashwin Mehta, Kristopher L. Arheart, Lora E. Fleming, Xuan Yang, Laura A. McClure, David J. Lee

**Affiliations:** 1 Department of Public Health Sciences, University of Miami, Miller School of Medicine, Miami, Florida; 2 Sylvester Comprehensive Cancer Center, University of Miami, Miller School of Medicine, Miami, Florida; 3 European Centre for Environment and Human Health, University of Exeter Medical School, Knowledge Spa, Royal Cornwall Hospital, Truro, Cornwall, United Kingdom.

## Abstract

**Introduction:**

Mindfulness-based practices can improve workers’ health and reduce employers’ costs by ameliorating the negative effect of stress on workers’ health. We examined the prevalence of engagement in 4 mindfulness-based practices in the US workforce.

**Methods:**

We used 2002, 2007, and 2012 National Health Interview Survey (NHIS) data for adults (aged ≥18 y, n = 85,004) to examine 12-month engagement in meditation, yoga, tai chi, and qigong among different groups of workers.

**Results:**

Reported yoga practice prevalence nearly doubled from 6.0% in 2002 to 11.0% in 2012 (*P* < .001); meditation rates increased from 8.0% in 2002 to 9.9% in 2007 (*P* < .001). In multivariable models, mindfulness practice was significantly lower among farm workers (odds ratio [OR] = 0.42; 95% confidence interval [CI], 0.21–0.83]) and blue-collar workers (OR = 0.63; 95% CI, 0.54–0.74) than among white-collar workers.

**Conclusion:**

Worker groups with low rates of engagement in mindfulness practices could most benefit from workplace mindfulness interventions. Improving institutional factors limiting access to mindfulness-based wellness programs and addressing existing beliefs about mindfulness practices among underrepresented worker groups could help eliminate barriers to these programs.

## Introduction

Over the last several decades, mindfulness-based interventions (MBIs) have gained wide recognition through such programs as Mindfulness-Based Stress Reduction (1), Mindfulness-Based Cognitive Therapy (2), Mindfulness-Based Relapse Prevention (3), and others. The effectiveness of MBIs for the treatment of difficult and chronic clinical problems (eg, chronic pain, mood disorders, substance abuse) (4–6), as well as for stress in healthy populations (7), has been well demonstrated. Mindfulness, the main therapeutic element of these programs, is defined as the intentional and nonjudgmental conscious awareness of the present moment (1). This quality of mind is used and developed through varied meditation techniques and through physical movements and martial arts traditions such as yoga and tai chi. A typical MBI program incorporates a combination of mindfulness meditation and mindful movement based primarily on yoga, with some inclusions of other mindfulness-based practices such as tai chi or qigong.

MBI implementation at the workplace takes many forms, ranging from employee wellness programs to leadership training. Growing evidence demonstrates the beneficial effects of mindfulness practices among workers, in terms of both physical symptoms (eg, pain) and mental well-being. For example, meditation interventions targeting workers are effective at reducing work-associated stress, depression, and anxiety among full-time Australian workers (8). In health care providers, mindfulness training reduced burnout, mood disturbances, and stress (9,10). Mindfulness training also showed improvements in mood and sleep quality among teachers (11).

Workplace stress is associated with many poor health outcomes, both mental and physical (12,13); workplace stress is linked with decreased productivity, increased occupational injury, and absenteeism (14,15), as well as with substantially higher medical expenditures among highly stressed employees (16). By helping employees manage stress better, mindfulness-based practices, whether formal or informal, can improve workers’ health, increase productivity, and reduce employers’ costs (17).

Rates of engagement in mindfulness-based practices among varying groups of workers are unknown. It is also unknown which worker subgroups have better access to such practices or could benefit from improved access to them. In this study, we examined the rates of engagement in common mindfulness-based practices in US workers and compared these rates for 4 major occupational categories.

## Methods

### Data

Since 1957, the National Center for Health Statistics has conducted the National Health Interview Survey (NHIS), a multipurpose and multistage probability survey of the US noninstitutionalized civilian population (www.cdc.gov/nchs/nhis). Information is collected yearly through the NHIS on its participants’ sociodemographic and health characteristics. In addition to the core components of NHIS, the Alternative Health/Complementary and Alternative Medicine Supplement was included in the survey during 2002, 2007, and 2012. The supplement was administered to all adult participants of NHIS, and it assessed lifetime and 12-month use of various complementary and alternative health practices, including such mindfulness-based techniques as meditation and mind-body exercise activities containing a mindfulness element, such as yoga, tai chi, and qigong. The final annual response rates for the Adult Alternative Health Supplement during the 3 years of its administration averaged 66.5% (range, 59.4%–73.7%) (18). We used the available NHIS Alternative Health/Complementary and Alternative Medicine Supplement data for adults (aged ≥18 y) to examine workers’ 12-month engagement in the following mindfulness-based practices: 1) meditation, 2) yoga, 3) tai chi, 4) qigong, or 5) any of the 4 practices.

### Variables

Participants were dichotomized on each outcome based on whether they reported engaging in each individual practice or any of the practices in the previous 12 months (yes/no). These were treated separately as outcomes to create 5 outcome variables: meditation, yoga, tai chi, qigong, and any of the 4 practices. The assessment question for meditation practice in 2002 and 2007 was altered substantially for 2012. During 2002 and 2007, participants were asked, “During the past 12 months, did you use meditation?” In 2012, this question was replaced with 3 questions, each of which asked “During the past 12 months, did you use . . . ” about the following practices: 1) mantra meditation, including transcendental meditation, relaxation response, and clinically standardized meditation; 2) mindfulness meditation, including Vipassana, Zen Buddhist meditation, mindfulness-based stress reduction, and mindfulness-based cognitive therapy; and 3) spiritual meditation including centering prayer and contemplative meditation. As a result, the 2012 meditation data are not comparable to those from 2002 and 2007; therefore, when modeling meditation or any of the 4 practices, only data from 2002 and 2007 were used.

Employment was assessed for the week before the interview. Participants who worked during that week and those who reported having a job or business to return to if they did not work were classified as employed. Occupation was assessed with the question, “What kind of work were you doing?” referencing the job the person reported having in the last week. NHIS classifies occupation based on the Standard Occupational Classification System Census codes (www.bls.gov/tus/iocodes.htm), which we collapsed into 4 categories of workers: 1) white collar, 2) blue collar, 3) service, and 4) farm (19).

When comparing the odds of practice across occupations, the following variables were included as potential confounders: educational level (less than a high school diploma [reference], high school diploma, and more than a high school diploma), sex, race/ethnicity (non-Hispanic white [reference], non-Hispanic black, Hispanic, Asian, other), age (continuous), and household income (as a ratio of income to current poverty level, treated as a continuous variable).

### Statistical analysis

Prevalence and 95% confidence intervals (CIs) were calculated, and trends over survey years were assessed using χ^2^ tests. The prevalence of mindfulness practices was compared among worker groups using logistic regression. For each of the 5 study outcomes, 3 nested models were fitted; model 1 adjusted for survey year only; model 2 adjusted for survey year and adjusted for age, sex, and race/ethnicity; and Model 3 adjusted for the variables in model 2 and adjusted for income and education levels. Because of the complex sampling design, all estimates were adjusted for unequal selection and clustering using sampling weights and stratification variables available in NHIS. The sampling weights used were those included in the adult core component of NHIS adjusted for the use of combined survey years, as specified by Botman and Jack (20).

## Results

A total of 85,004 US adults aged 18 years or older, among whom 50,343 were employed (representing approximately 131 million US workers), answered questions about engaging in various mindfulness practices in the 3 survey periods. The characteristics of the study sample are presented in Table 1.

**Table 1:** Characteristics of Adult Participants (Aged ≥18 y), National Health Interview Survey Alternative Medicine Supplement (N = 85,004), 2002, 2007, and 2012

Characteristic	N	Participants, % (SE)				
		Unemployed (n = 34,661)	White-Collar Worker (n = 29,418)	Service Worker (n = 9,242)	Farm Worker (n = 716)	Blue-Collar Worker (n = 10,967)
**Education level**						
<High school diploma	15,076	23.19 (0.33)	3.54 (0.13)	18.53 (0.57)	45.34 (2.32)	21.56 (0.55)
High school diploma	23,278	31.54 (0.33)	18.24 (0.29)	32.27 (0.68)	30.85 (1.94)	42.51 (0.57)
>High school diploma	46,135	45.27 (0.42)	78.22 (0.30)	49.20 (0.70)	23.81 (1.67)	35.93 (0.60)
**Race/ethnicity**						
Hispanic	14,659	12.41 (0.28)	8.98 (0.23)	20.27 (0.57)	40.67 (2.71)	19.49 (0.52)
Non-Hispanic white	53,099	70.26 (0.41)	75.46 (0.36)	57.70 (0.67)	54.01 (2.57)	65.28 (0.58)
Non-Hispanic black	12,430	12.16 (0.27)	9.58 (0.24)	16.36 (0.47)	3.82 (0.42)	11.48 (0.37)
Non-Hispanic Asian	4,007	4.16 (0.15)	5.37 (0.17)	4.21 (0.24)	1.02 (0.36)	2.77 (0.20)
Other	809	1.01 (0.13)	0.61 (0.05)	1.47 (0.21)	0.48 (0.26)	0.98 (0.13)
**Sex**						
Male	37,343	39.29 (0.36)	44.08 (0.39)	41.91 (0.66)	82.02 (1.35)	84.88 (0.41)
Female	47,661	60.71 (0.36)	55.92 (0.39)	58.09 (0.66)	17.98 (1.35)	15.12 (0.41)
**Survey year**						
2002	29,878	30.10 (0.42)	32.52 (0.47)	25.45 (0.57)	54.12 (2.56)	34.20 (0.65)
2007	22,054	32.23 (0.46)	33.65 (0.49)	35.51 (0.73)	20.32 (2.31)	34.38 (0.70)
2012	33,072	37.67 (0.40)	33.83 (0.37)	39.04 (0.74)	25.57 (2.37)	31.43 (0.62)
**Age^a^**	—	54.06 (0.19)	41.77 (0.11)	39.04 (0.21)	38.98 (0.56)	40.80 (0.17)

Abbreviation: —, not applicable.

a Mean (SE) age was 46 (0.11) years.

The prevalence of practices in the overall worker population ranged from 0.3% (qigong in 2002 and 2007) to 11.0% (yoga in 2012) (Table 2). Across worker groups, between 2.2% (farm workers in 2002) and 18.2% (white-collar workers in 2007) reported engaging in at least 1 of the practices; approximately 9% to 12% of the unemployed did so. The prevalence of yoga practice increased significantly over the 3 survey years from 6.0% in 2002 to 11.0% in 2012 (*P* < .001). From 2002 to 2007, the prevalence of engagement in meditation increased significantly, from 8.0% to 9.9%, and prevalence of engagement in any of the 4 practices increased significantly, from 11.7% to 14.3% (*P* < .001).

**Table 2:** Prevalence of 12-Month Engagement in Mindfulness-Based Practices Among Adults (Aged ≥18 y), by Employment Status and Occupation, National Health Interview Survey Alternative Medicine Supplement, 2002, 2007, and 2012

Practice/Occupation Status	2002	2007	2012^a^
	% (Standard Error)		
**Yoga**			
Unemployed	3.71 (0.20)	4.75 (0.29)	6.59 (0.30)
All workers	5.95 (0.21)	6.85 (0.29)	11.00 (0.34)
White-collar workers	8.26 (0.32)	9.33 (0.41)	14.22 (0.48)
Service workers	4.53 (0.47)	6.29 (0.60)	10.67 (0.66)
Farm workers	1.50 (0.70)	—	2.33 (1.69)
Blue-collar workers	1.72 (0.18)	1.39 (0.26)	3.07 (0.33)
**Meditation**			
Unemployed	6.89 (0.30)	8.76 (0.41)	3.64 (0.22)
All workers	8.01 (0.25)	9.93 (0.32)	4.41 (0.21)
White-collar workers	9.79 (0.34)	12.25 (0.48)	5.22 (0.29)
Service workers	7.65 (0.60)	9.27 (0.65)	5.36 (0.51)
Farm workers	1.59 (0.79)	5.79 (2.46)	0.19 (0.19)
Blue-collar workers	4.91 (0.38)	5.67 (0.61)	1.70 (0.25)
**Tai chi**			
Unemployed	1.35 (0.14)	0.99 (0.15)	1.03 (0.10)
All workers	1.23 (0.10)	1.08 (0.10)	1.21 (0.10)
White-collar workers	1.55 (0.14)	1.23 (0.15)	1.38 (0.15)
Service workers	1.22 (0.23)	1.17 (0.24)	1.21 (0.22)
Farm workers	—		
Blue-collar workers	0.56 (0.13)	0.72 (0.17)	0.86 (0.18)
**Qigong**			
Unemployed	0.25 (0.05)	0.34 (0.09)	0.28 (0.05)
All workers	0.27 (0.04)	0.26 (0.05)	0.39 (0.05)
White-collar workers	0.36 (0.07)	0.28 (0.06)	0.49 (0.08)
Service workers	0.08 (0.05)	0.30 (0.12)	0.43 (0.10)
Farm workers	—		
Blue-collar workers	0.16 (0.07)	0.14 (0.08)	0.12 (0.06)
**Any of the 4 practices^b^**			
Unemployed	9.48 (0.32)	11.74 (0.46)	9.32 (0.35)
All workers	11.68 (0.31)	14.34 (0.37)	13.64 (0.37)
White-collar workers	15.05 (0.44)	18.16 (0.53)	17.21 (0.51)
Service workers	10.05 (0.69)	13.47 (0.80)	13.74 (0.79)
Farm workers	2.20 (0.90)	5.73 (2.43)	2.42 (1.71)
Blue-collar workers	5.83 (0.39)	6.72 (0.63)	4.52 (0.40)

Abbreviation: —, data not available.

a 2012 Prevalences of meditation and all 4 practices are not comparable to previous years because of substantial changes in the meditation questions.

b Yoga, meditation, tai chi, or qigong.

In Model 1, after adjustment for survey year, the unemployed and all other worker groups were significantly less likely to engage in meditation, yoga, or any of the 4 practices than were white-collar workers (Table 3). In 2002 and 2007, the odds of engaging in any of the 4 practices ranged from 0.17 (95% CI, 0.09–0.32) for farm workers to 0.67 (95% CI, 0.60–0.75) for service workers, compared with white-collar workers. These significant differences remained when additionally controlling for age, sex, and race/ethnicity (model 2); service, farm, and blue-collar workers were significantly less likely than white-collar workers to engage in meditation or yoga (Table 3). Blue-collar workers were also less likely than white-collar workers to practice tai chi (OR = 0.55; 95% CI, 0.41–0.75) or qigong (OR = 0.41; 95% CI, 0.22–0.77).

**Table 3:** Multivariable Logistic Regression Results, Odds of 12-Month Engagement in Mindfulness-Based Practices Among Adult (Aged ≥18 y) Worker Subgroups, National Health Interview Survey Alternative Medicine Supplement, 2002, 2007, and 2012^a^

Characteristic	Model 1	Model 2	Model 3
	Odds Ratio (95% Confidence Interval)		
**Meditation in Previous 12 Months (2002 and 2007 Data Only)**			
**Employment/occupation**			
White-collar worker	1 [Reference]		
Unemployed	0.69 (0.63–0.75)	0.75 (0.68–0.83)	1.00 (0.89–1.13)
Service worker	0.81 (0.65–0.85)	0.76 (0.67–0.87)	0.95 (0.81–1.11)
Farm worker	0.21 (0.12–0.40)	0.30 (0.15–0.58)	0.54 (0.26–1.09)
Blue-collar worker	0.43 (0.39–0.53)	0.52 (0.44–0.60)	0.71 (0.59–0.85)
**Age**		0.99 (0.99–1.00)	1.00 (0.99–1.00)
**Sex**			
Female	1 [Reference]		
Male		0.80 (0.74–0.86)	0.77 (0.71–0.84)
**Race/ethnicity**			
Non-Hispanic white	1 [Reference]		
Hispanic		0.58 (0.50–0.67)	0.71 (0.60–0.85)
Non-Hispanic black		0.93 (0.83–1.05)	0.98 (0.86–1.11)
Asian		1.03 (0.86–1.24)	1.00 (0.81–1.23)
Other		1.52 (1.03–2.25)	1.55 (1.00–2.40)
**Education level**			
<High school diploma	1 [Reference]		
High school diploma			1.61 (1.31–1.98)
>High school diploma			3.58 (2.97–4.31)
**Income-to-poverty ratio^b^**			1.01 (1.00–1.02)
**Yoga in Previous 12 Months**			
**Employment/occupation**			
White-collar worker	1 [Reference]		
Unemployed	0.44 (0.41–0.48)	0.56 (0.51–0.60)	0.86 (0.77–0.95)
Service worker	0.66 (0.59–0.74)	0.66 (0.60–0.74)	0.94 (0.83–1.06)
Farm worker	0.13 (0.06–0.29)	0.21 (0.09–0.48)	0.39 (0.14–1.03)
Blue-collar worker	0.18 (0.15–0.21)	0.29 (0.25–0.34)	0.46 (0.39–0.55)
**Age**		0.97 (0.97–0.98)	0.97 (0.97–0.98)
**Sex**			
Female	1 [Reference]		
Male		0.36 (0.33–0.39)	0.34 (0.31–0.38)
**Race/ethnicity**			
Non-Hispanic white	1 [Reference]		
Hispanic		0.45 (0.40–0.50)	0.65 (0.57–0.74)
Non-Hispanic black		0.44 (0.40–0.49)	0.55 (0.49–0.62)
Asian		1.19 (1.03–1.36)	1.17 (1.00–1.36)
Other		0.92 (0.65–1.30)	1.04 (0.73–1.48)
**Education level**			
<High school diploma	1 [Reference]		
High school diploma			1.67 (1.30–2.14)
>High school diploma			4.51 (3.53–5.77)
**Income-to-poverty ratio^b^**			1.06 (1.05–1.07)
**Tai Chi in Previous 12 Months**			
**Employment/occupation**			
White-collar worker	1 [Reference]		
Unemployed	0.80 (0.67–0.97)	0.80 (0.65–0.98)	1.12 (0.87–1.43)
Service worker	0.88 (0.67–1.14)	0.92 (0.71–1.20)	1.20 (0.91–1.58)
Farm worker	—c		
Blue-collar worker	0.51 (0.38–0.68)	0.55 (0.41–0.75)	0.88 (0.63–1.22)
**Age**		1.00 (1.00–1.01)	1.01 (1.00–1.01)
**Sex**			
Female	1 [Reference]		
Male		0.97 (0.83–1.13)	0.94 (0.79–1.12)
**Race/ethnicity**			
Non-Hispanic white	1 [Reference]		
Hispanic		0.62 (0.48–0.79)	0.76 (0.58–1.00)
Non-Hispanic black		0.99 (0.79–1.24)	1.06 (0.84–1.35)
Asian		2.43 (1.87–3.15)	2.28 (1.72–3.04)
Other		2.36 (1.40–3.98)	2.91 (1.72–4.91)
**Education level**			
<High school diploma	1 [Reference]		
High school diploma			1.64 (1.04–2.59)
>High school diploma			4.51 (3.04–6.71)
**Income-to-poverty ratio^b^**			1.01 (0.99–1.04)
**Qigong in Previous 12 Months**			
**Employment/occupation**			
White-collar worker	1 [Reference]		
Unemployed	0.77 (0.55–1.07)	0.75 (0.51–1.10)	1.04 (0.64–1.68)
Service worker	0.77 (0.50–1.19)	0.86 (0.55–1.33)	1.21 (0.77–1.88)
Farm worker	—c		
Blue-collar worker	0.38 (0.21–0.71)	0.41 (0.22–0.77)	0.61 (0.29–1.26)
**Age**		1.01 (1.00–1.01)	1.01 (1.00–1.02)
**Sex**			
Female	1 [Reference]		
Male		1.05 (0.79–1.40)	0.98 (0.71–1.36)
**Race/ethnicity**			
Non-Hispanic white	1 [Reference]		
Hispanic		0.57 (0.32–1.01)	0.71 (0.36–1.40)
Non-Hispanic black		0.56 (0.35–0.89)	0.56 (0.33–0.96)
Asian		1.81 (1.12–2.92)	1.80 (1.07–3.05)
Other		1.72 (0.58–5.14)	2.19 (0.73–6.54)
**Education level**			
<High school diploma	1 [Reference]		
High school diploma			1.33 (0.49–3.56)
>High school diploma			4.70 (1.90–11.59)
**Income-to-poverty ratio^b^**			1.02 (0.97–1.06)
**Any of the 4 Practices^d^ in Previous 12 Months (2002 and 2007 Data Only)**			
**Employment/occupation**			
White-collar worker	1 [Reference]		
Unemployed	0.60 (0.55–0.64)	0.69 (0.63–0.74)	0.97 (0.88–1.08)
Service worker	0.67 (0.60–0.75)	0.69 (0.62–0.78)	0.90 (0.79–1.03)
Farm worker	0.17 (0.09–0.32)	0.24 (0.13–0.44)	0.42 (0.21–0.83)
Blue-collar worker	0.34 (0.30–0.38)	0.43 (0.38–0.49)	0.63 (0.54–0.74)
**Age**		0.99 (0.99–0.99)	0.99 (0.99–0.99)
**Sex**			
Female	1 [Reference]		
Male		0.63 (0.58–0.67)	0.60 (0.55–0.65)
**Race/ethnicity**			
Non-Hispanic white	1 [Reference]		
Hispanic		0.51 (0.46–0.58)	0.65 (0.57–0.75)
Non-Hispanic black		0.75 (0.68–0.83)	0.81 (0.73–0.91)
Asian		1.23 (1.06–1.44)	1.19 (1.00–1.41)
Other		1.4 (0.97–2.01)	1.47 (0.99–2.19)
**Education level**			
<High school diploma	1 [Reference]		
High school diploma			1.56 (1.31–1.86)
>High school diploma			3.78 (3.20–4.46)
**Income-to-poverty ratio^b^**			1.02 (1.01–1.03)

a Model 1 adjusted for survey year only; Model 2 adjusted for survey year, age, sex, and race/ethnicity; and Model 3 adjusted for survey year, age, sex, race/ethnicity, income, and education level.

b 14 Ordinal categories.

c Data not presented because of small sample size.

d Yoga, meditation, tai chi, or qigong.

After additionally adjusting for education and income level (model 3), the odds of engaging in any of the 4 practices were significantly lower in farm workers (OR = 0.42; 95% CI, 0.21–0.83) and blue-collar workers (OR = 0.63; 95% CI, 0.54–0.74) than among white-collar workers; no significant difference was seen for service workers (OR = 0.90; 95% CI, 0.79–1.03). In model 3, blue-collar workers were also significantly less likely than white-collar workers to engage in meditation (OR = 0.71; 95% CI, 0.59–0.85) or yoga (OR = 0.46; 95% CI, 0.39–0.55); however, no significant differences were found for other types of workers for these practices.

## Discussion

In this study, we examined the rates of 12-month engagement in 4 common mindfulness-based practices (meditation, yoga, tai chi, and qigong) in US workers, and we compared these rates among major occupational groups, using nationally representative data. To our knowledge, this is the first study to characterize the prevalence of engagement in these practices in the workforce. We found that approximately 12% to 14% of workers and 9% to 12% of the unemployed reported having engaged in at least 1 of these practices within the past year. Over the decade of survey data available, the rates of engagement in some practices (eg, yoga, meditation) increased; rates of yoga practice among workers rose almost twofold between 2002 and 2012. However, the rates of engagement in the lesser-known practices of tai chi and qigong did not substantially change during this period. The rates of engagement in yoga in the general population have risen steadily during the past 2 decades (21), likely being driven by a combination of factors, including increased public awareness of health benefits (22,23), health care provider recommendations to their patients (23,24), and the growth in the number of yoga studios and other classroom-based venues available for practice (21,23). Furthermore, the clinical success and dissemination of MBI programs, such as Mindfulness-Based Stress Reduction and its derivatives, could explain some of the increase in the rates of meditation practice engagement.

We found substantial variation in the rates of mindfulness practice engagement across occupations. For example, white-collar workers were more likely than all other workers to engage in yoga or meditation, and they were more likely than blue-collar workers to engage in tai chi or qigong. Most of these differences, however, most likely can be attributed to differences in household income and education level. After controlling for these 2 factors, blue-collar workers were still less likely than white-collar workers to engage in meditation or yoga, and farm workers continued to be less likely to engage in any of the 4 practices.

Sociodemographic factors (eg, lower educational attainment, male sex) may be a challenge to wider MBI implementation among US workers (25). Additionally, there seems to be a lack of engagement in mindfulness practices among blue-collar workers and farm workers beyond what can be explained by sociodemographic factors. Moreover, lower rates of meditation or yoga engagement among blue-collar workers, even after controlling for sociodemographic factors, may indicate differences in beliefs about the value of these practices among these workers (26). Additionally, more leisure time, more access to workplace and other resources, and more regular work schedules may provide more opportunity to practice mindfulness activities among white-collar workers relative to other worker groups.

One study limitation was that the location of mindfulness practice, such as in the workplace itself, was not assessed. In recent years, with the development of the National Institute for Occupational Safety and Health Total Worker Health (TWH) program (www.cdc.gov/niosh/twh), the emphasis of workplace health promotion has been shifting toward work–life balance and overall worker well-being. The TWH program encourages measures that target both work-related and nonwork-related factors affecting worker health. Workplace MBIs can address this shift in emphasis. A review of complementary therapies offered in the workplace shows that mindfulness-based and meditation-based interventions were the most effective at improving workers’ psychological well-being (27).

MBIs are integrated into worksites in many different ways, including web-based programs, yoga or meditation classes, and full mindfulness-based stress reduction programs (17,28). These MBIs each have their own challenges, which can include cost, time required, expertise, and participant retention. Despite these challenges, MBIs offer substantial advantages, because they can provide workers with skills for coping with stress, whether or not it is work-related, and for improving mood management and emotional regulation (29). Mindfulness practice can also increase workers’ resilience, thereby enabling them to better deal with stress while preventing burnout, which is especially true for high-stress occupations. MBIs also have been effective in health behavior modification, such as smoking cessation and substance abuse prevention measures (4,30). Reducing these risky behaviors reduces chronic health conditions, such as heart disease and cancer, resulting in an overall healthier workforce. Some other potential benefits of workplace MBIs are increased productivity, memory, creativity, focus, impulse control, and emotional intelligence (9).

We could find no intervention studies in the literature which focused on blue-collar or farm workers. Given the low prevalence of these practices noted in this study, there is a pressing need for the development of interventions targeting these occupational groups. These types of workplace settings may present unique implementation challenges compared with similar interventions that target worksites with white-collar workers.

Our finding of high and increasing rates of exposure to mindfulness practices among US workers is encouraging. Approximately 1 in 7 workers report engagement in some form of mindfulness-based activity, and these individuals can bring awareness of the benefit of such practices into the workplace. Identifying workers who do engage in mindfulness activities and involving them in the promotion of awareness about these in the workplace could increase acceptance of MBIs among occupations that underrepresented among mindfulness practitioners. Managers should take into account and identify such individuals when planning the implementation of MBIs in the workplace. Institutional factors, such as lack of funding or lack of work time for workplace opportunities, that prevent equal access to various health-promotion measures as well as individual beliefs preventing engagement in mindfulness practices should be addressed to make these practices available to all workers.

Although overall rates of engagement in mindfulness practices, such as yoga and meditation, are increasing in the workforce, variation in rates of engagement in mindfulness practices exists across occupational groups. Mindfulness practice can address multiple workplace wellness needs, benefiting both employees and employers. Development of workplace mindfulness programs should target occupational groups that have low rates of engagement in such practices (ie, blue-collar and farm workers), placing emphasis on men and on socioeconomically disadvantaged subgroups within these occupations. This development should be done both by improving institutional factors that limit access to mindfulness-based wellness programs and addressing existing beliefs about mindfulness practices among underrepresented worker groups.
